# A single extinction-based treatment with N-Acetylcysteine produces long-term reduction in cocaine relapse

**DOI:** 10.1038/s41398-026-03954-2

**Published:** 2026-03-19

**Authors:** Shihao Huang, Zhihao Song, Cuijie Shi, Dan Tao, Jing Wen, Yisi Chen, Yixiao Luo

**Affiliations:** 1 The First-Affiliated Hospital of Hunan Normal University, Hunan Province People’s Hospital, Changsha, 410081 China; 2 Department of Neurobiology, School of Basic Medical Sciences, National Institute on Drug Dependence, Peking University, Beijing, China; 3 Key Laboratory for Birth Defects Research and Prevention of the National Health Commission, Hunan Provincial Maternal and Child Health Care Hospital, Changsha, China; 4 Department of Neurosurgery, Xiangya Hospital, Central South University, 410008 Changsha, Hunan China; 5 College of Forensic Medicine, Hebei Medical University, Shijiazhuang, China; 6 School of Medicine, Hunan Normal University, Changsha, China

**Keywords:** Addiction, Long-term memory

## Abstract

Cocaine addiction is characterized by high relapse rates associated with glutamate dysregulation, presenting significant challenges for long-term treatment. N-acetylcysteine (NAC) has shown promise in preventing drug relapse by normalizing glutamate function, potentially mediated by glutamate 2/3 (mGlu2/3) receptor activation. This study investigated the therapeutic potential of NAC in reducing cocaine-seeking behavior using the self-administration model. After establishing stable cocaine self-administration and a 7-day abstinence period, rats received a single dose of NAC (100 mg/kg, i.p.) 30 min before the first extinction training session. NAC significantly reduced cocaine-seeking behavior on the first day of extinction but not in subsequent extinction sessions. Following extinction, tests for cue-induced reinstatement and spontaneous recovery were conducted. Results showed that NAC administration on the first day of extinction effectively reduced cocaine-seeking during the reinstatement test, with effects lasting at least 28 days. The mGlu2/3 antagonist LY341495 (1 mg/kg) fully blocked this enduring suppression of reinstatement without altering the immediate decrease in drug-seeking observed on the first day of extinction. Additionally, NAC administration on the initial extinction day also reduced context-induced reinstatement of cocaine-seeking behavior. These results indicate that NAC exerts its anti-relapse effects via mGlu2/3 receptors. A single NAC treatment combined with extinction training can produce lasting suppression of relapse, highlighting its therapeutic promise for addiction treatment.

## Introduction

Cocaine use disorder (CUD) is marked by a progression from voluntary, recreational use to compulsive drug-taking behavior, accompanied by addiction-related phenomena such as craving and paranoia [1, 2]. A defining characteristic of CUD is the high propensity for relapse, even following extended periods of abstinence, which underscores the urgent need for effective anti-relapse pharmacotherapies [3, 4]. Current treatments primarily rely on psychosocial approaches—contingency-based reinforcement and cognitive-behavioral therapy, which typically produce only modest effect sizes [5]. To date, no pharmacotherapy has demonstrated consistent efficacy and safety for CUD, and the lack of FDA-approved treatments leaves relapse prevention as a significant clinical challenge [6, 7].

Extensive preclinical and clinical evidence implicates maladaptive glutamate signaling in cocaine addiction and relapse [8–10]. As the primary excitatory neurotransmitter in the brain, glutamate drives corticolimbic circuits—the glutamatergic projections from the prefrontal cortex to the nucleus accumbens, which are central to the development, persistence, and reinstatement of drug-seeking behavior and craving [11, 12]. Prolonged cocaine self-administration lowers baseline extracellular glutamate levels while allowing transient glutamate surges during reinstatement; in part due to downregulation of the cystine–glutamate exchanger(system xc⁻) and the astrocytic glutamate transporter GLT-1, both of which impair synaptic clearance [10]. This dysregulation disrupts synaptic homeostasis, facilitating drug-seeking responses when subjects encounter cocaine-associated cues or contexts [13, 14]. Therefore, pharmacological targeting of glutamate-related mechanisms in the central nervous system represents a promising strategy for developing effective treatments for CUD [15].

N-acetylcysteine (NAC), a prodrug of cystine, re-establishes basal glutamatergic tone by providing cystine for system xc⁻, thereby normalizing extracellular glutamate levels and activating presynaptic metabotropic glutamate receptors 2/3 (mGluR2/3) [16–18]. This mechanism reverses drug-induced synaptic alterations and attenuates relapse behaviors in animal models. This candidacy is supported by evidence that persistent glutamatergic dysregulation is observed across models of cocaine, heroin, alcohol, and nicotine relapse [19–22]. LaRowe et al. first reported in an open-label pilot study that NAC reduced craving and cocaine use in individuals with CUD [14]. A 2012 crossover MRS study showed that 7 days of NAC normalized elevated dorsal anterior cingulate cortex glutamate in cocaine-dependent subjects, paralleling reductions in craving and attentional bias [23]. Subsequent work by Levi Bolin and Woodcock further demonstrated that NAC attenuates cocaine-cue–induced attentional bias, reduces cocaine self-administration and subsequent cocaine-seeking behavior through modulation of ACC glutamate [24, 25]. However, clinical trials of NAC have shown limited efficacy, suggesting its role may be most beneficial as an adjunct to standard treatment or psychotherapeutic interventions [26]. Moreover, the relapse-preventing effects of NAC are believed to arise chiefly from the restoration of extrasynaptic glutamate to a physiological level, thereby preferentially activating mGluR2/3 receptors [27]. Activation of mGluR2/3 reduces synaptic glutamate release and reverses cocaine-induced synaptic potentiation, effectively inhibiting both cue- and drug-primed reinstatement in chronic treatment paradigms [28–30]. Importantly, acute NAC administration also prevents cocaine-induced elevations of extracellular glutamate in the nucleus accumbens, highlighting its potential for relapse prevention [31, 32]. Consequently, there is a need to incorporate NAC into therapeutic regimens, which may provide a more effective strategy for preventing relapse.

Extinction training—repeated, non-reinforced exposure to drug-paired cues—suppresses conditioned drug-seeking and forms new “no-drug” memories but does not erase original drug memories, as evidenced by spontaneous recovery, renewal, and reinstatement phenomena [33]. Accumulating evidence suggests that extinction learning engages glutamatergic signaling pathways, particularly within cortico-limbic circuits implicated in drug memory and behavioral flexibility [34]. Furthermore, acute NAC administration reduces craving for drug-related cues in cocaine-dependent individuals [14] and facilitates the extinction of fear memory [35]. Therefore, we hypothesized that a single NAC treatment combined with extinction training might restore glutamatergic homeostasis and strengthen the new “no-drug” memory trace, thereby reducing relapse vulnerability.

Using a rat intravenous self-administration model, we administered NAC 30 min before the first extinction training and assessed its effects on cue- or drug-induced reinstatement, spontaneous recovery, and context-induced relapse of cocaine-seeking behavior. To investigate the role of mGluR2/3 receptors, we also assessed whether their pharmacological blockade during NAC treatment would diminish its sustained anti-relapse effectiveness.

## Method and materials

### Subject

Adult male Sprague-Dawley rats (260–280 g; Tianqin Laboratory Animal Technology Co., Ltd., China) were housed individually under controlled conditions with ad libitum access to food and water. Animals were kept on a 12 h reversed light/dark schedule (lights off from 08:00–20:00), with all procedures carried out during the light phase. The Local Committee approved all experimental procedures on Animal Care, Use, and Protection of the Hunan Normal University (D2024400).

### Drugs

Prior to each experiment, N-acetylcysteine (NAC; 10 or 100 mg/kg; Sigma, St. Louis, MO, United States) and cocaine hydrochloride were dissolved in 0.9% saline. VEH groups received saline vehicle at equivalent volumes. LY341495 (Tocris, Bristol, UK) was prepared in 0.9% saline and administered via intraperitoneal injection at a dose of 1 mg/kg.

### Intravenous surgery

All surgical procedures were conducted under aseptic conditions, with anesthesia induced using 4–5% isoflurane and maintained at 1.5–2%, after which rats were positioned in a stereotaxic apparatus. After full anesthesia was achieved, incisions were made on the chest and back. A catheter was inserted into the right jugular vein and advanced to the entrance of the right atrium; the catheter was then tunneled subcutaneously to exit on the dorsal aspect between the scapulae [36, 37]. Following surgery, rats were allowed to recover for 5–7 days and administered daily doses of penicillin (0.5 mg/kg) to prevent infection.

### Cocaine self-administration training

The experimental chambers (AniLab, China) were fitted with two nosepoke holes at identical elevations. An entry into the left port (active nosepoke) triggered a delivery of cocaine (0.75 mg/kg per infusion, i.v.) accompanied by a 5 s compound cue consisting of a tone and a light. Responses in the right nosepoke (inactive nosepoke) port had no programmed consequence. The apparatus automatically logged both nosepoke counts and drug infusions.

Self-administration sessions lasted for 3 h each day, with a 5 min break every hour, and were conducted over 10 consecutive days. Drug infusions were delivered under a fixed-ratio 1 (FR1) reinforcement schedule, with each administration followed by a 40 s timeout period. Each session commenced with the house light illuminated, which remained on until the first active nosepoke response, after which the light was extinguished for 40 s before being reactivated. To minimize the likelihood of overdose-induced mortality, the number of infusions permitted per hour was limited to 20. Rats were excluded from further analysis if their intravenous catheters became occluded or showed evidence of leakage during the training phase, as specified by the predefined exclusion criteria.

### Withdrawal

Following the self-administration phase, rats underwent 7 days of forced abstinence in their home cages, during which neither cocaine exposure nor behavioral training occurred. Animals were regularly weighed and handled throughout this period.

### Nosepoke extinction

During extinction training, rats were reintroduced to the original self-administration chambers, where active nosepoke responses produced no outcomes—neither cocaine infusion nor the associated tone-light cue was provided. Extinction training continued daily until the number of active nosepoke dropped below 20% of the average count observed during the final three days of cocaine self-administration for at least two consecutive sessions.

### Cue-induced reinstatement test

In the same self-administration context, the number of nosepoke into the active and inactive operanda was measured over 1 h. All parameters matched the training phase except that active nosepoke did not result in cocaine injections. Instead, each active response triggered the previously paired tone-light cue only. Notably, the line previously used for cocaine infusion was not connected during the test session.

### Cue extinction

During extinction, rats were returned to the original training chambers. Active nosepoke elicited the cocaine-associated tone-light cues but no cocaine infusion, while inactive nosepoke produced no programmed outcomes.

### Drug-induced reinstatement test

Before the test session, rats were administered cocaine (10 mg/kg, i.p.) instead of exposure to the tone-light cues. Five minutes later, rats were returned to the self-administration context. All other test parameters remained consistent with those of the cue-induced reinstatement procedure.

### Context-induced reinstatement test

Rats were returned to the training chambers, where nosepoke responses had no programmed consequences, and no cues were presented. All remaining conditions were kept consistent with the self-administration phase.

### Spontaneous recovery test

Following a 28-day withdrawal, the rats were returned to their original chambers. Active nosepoke triggered the same light cue as during the training phase, but without cocaine infusion. Both active and inactive nosepoke responses were recorded.

### Open field test

The experiment took place in an acrylic chamber (100 × 100 × 50 cm³) with the floor segmented into 16 equal squares. The central four squares constituted the center zone, while the remaining twelve formed the peripheral zone. Each rat was positioned in the center and permitted to explore freely for 5 min. Measurements included time spent in the center, total distance, and locomotor speed. To minimize olfactory interference, the chamber was thoroughly cleaned with 75% ethanol and then wiped with water between trials.

### Statistical analyses

Data in this study were analyzed using GraphPad Prism v8.3 (GraphPad Software, USA). For simple comparisons, Student’s t-test was applied. Experiments with multiple groups were assessed by one-way or two-way analysis of variance (ANOVA), followed by Bonferroni or Tukey’s post hoc tests, as appropriate. Statistical significance was defined as two-tailed α = 0.05. All data are presented as mean ± SEM.

## Results

Total cocaine intake (mg/kg) during self-administration training was comparable across all experimental groups, with no significant differences observed (Supplemental Fig. 1). Comprehensive statistical results for all experiments are provided in Supplemental Table 1.

### Experiment 1: Pre-treatment with NAC prior to extinction day 1 reduces both cue- and drug-induced reinstatement of cocaine-seeking behavior

**Fig. 1:**
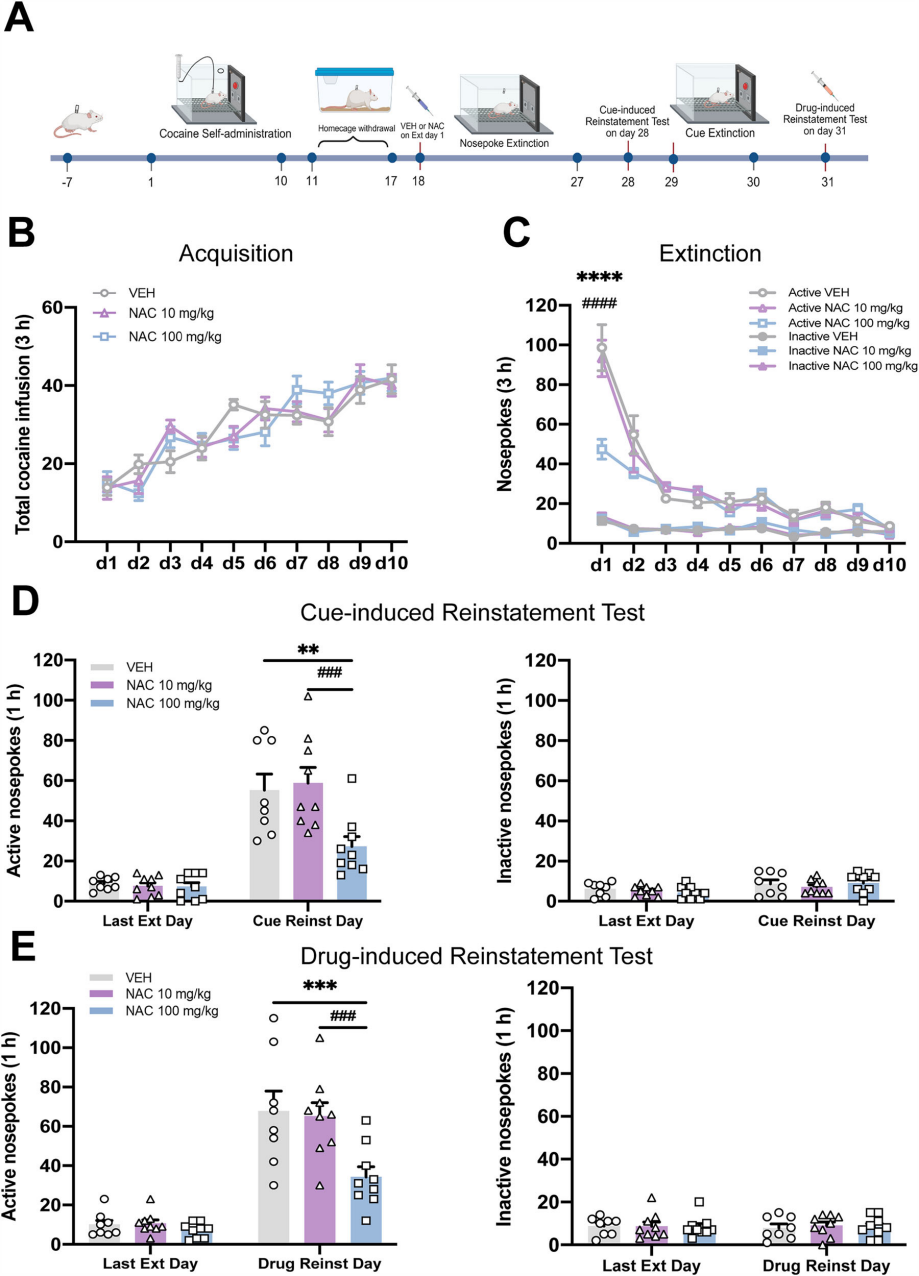
Pre-treatment with NAC prior to extinction day 1 reduces both cue- and drug-induced reinstatement of cocaine-seeking behavior. **A** Experimental timeline. After 10-day cocaine self-administration and 7-day homecage withdrawal period, NAC (10 or 100 mg/kg) or VEH were administered intraperitoneally 30 min prior to extinction day 1. After 10-day extinction training, rats underwent a cue-induced reinstatement test on day 28, followed by cue extinction sessions on days 29 and 30, and a drug-induced reinstatement test on day 31. **B** The mean number of cocaine infusions obtained during the acquisition of cocaine self-administration training. **C** The mean number of active and inactive nosepoke during the extinction training. **D** Active and inactive nosepoke responses recorded during the last extinction training and the cue-induced reinstatement test. ○, △, and □ represent the performance of rats administered VEH, 10 mg/kg NAC, and 100 mg/kg NAC, respectively. **E** Active and inactive nosepoke responses recorded during the last extinction training and the drug-induced reinstatement test. **P < 0.01, ***P < 0.001, ****P < 0.0001, comparing VEH to NAC 100 mg/kg; ^###^*P* < 0.001, ^####^*P* < 0.0001, comparing NAC 10 mg/kg to NAC 100 mg/kg. Active VEH = Active nosepoke in the VEH group. Inactive VEH = Inactive nosepoke in the VEH group. Last Ext Day = Last Extinction Day. Cue Reinst Day = Cue-induced Reinstatement Test Day. Drug Reinst Day = Drug-induced Reinstatement Test Day. Data are expressed as the mean ± SEM. Details of the statistical analyses are shown in Supplemental Table 1.

After cocaine self-administration and a 7-day withdrawal period in the home cage, rats were administered a single dose of NAC (10 or 100 mg/kg, i.p.) or vehicle (VEH) 30 min before the initial extinction session. Cue-induced reinstatement testing was conducted the day after the final extinction session. This was followed by two additional days of cue extinction, and a drug-priming reinstatement test was conducted the next day (Fig. 1A). Consistent with previous studies [38, 39], rats developed stable operant nosepoke behavior after 10 days of self-administration training, with no notable group differences detected (Fig. 1B). A two-way repeated-measures ANOVA on the extinction data identified a significant interaction between treatment × extinction day in relation to active nosepoke responses (*F*_*treatment* *×* *time*_(18,207) = 4.726*, P* < 0.0001). Post-hoc analysis indicated that rats administered NAC at 100 mg/kg exhibited significantly lower cocaine-seeking behavior on the first extinction day compared to those in the VEH or NAC 10 mg/kg groups (Fig. 1C. VEH *vs*. NAC 100 mg/kg, *P* < 0.0001*;* NAC 10 mg/kg *vs*. NAC 100 mg/kg, *P* < 0.0001). We also found that NAC 100 mg/kg attenuated reinstatement of cocaine-seeking behavior triggered by either drug-associated cues or cocaine priming (Fig. 1D, E. Cue-induced reinstatement, *F*_*treatment* _(2,23) = 6.398*, P* = 0.0062; Drug-induced reinstatement, *F*_*treatment* _(2,23) = 7.758*, P* = 0.0027), while no group differences were found in the inactive nosepoke responses (*P* > 0.1). Collectively, these findings suggest that a single NAC injection paired with extinction training exerts anti-relapse effects in cocaine-seeking behavior.

### Experiment 2: Pre-treatment with NAC prior to extinction day 1 decreases subsequent spontaneous recovery of cocaine-seeking behavior

**Fig. 2:**
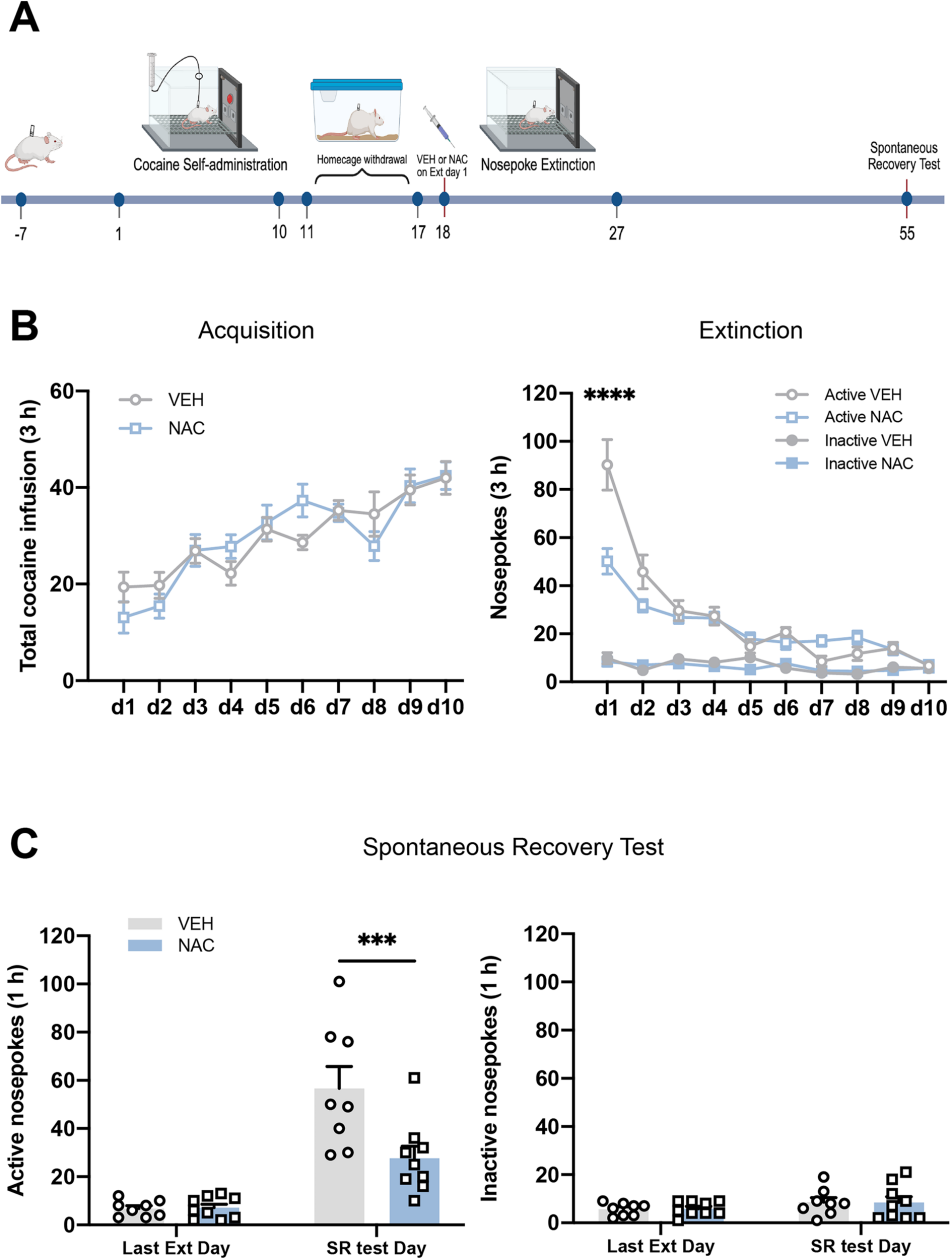
Pre-treatment with NAC prior to extinction day 1 decreases subsequent spontaneous recovery of cocaine-seeking behavior. **A** Experimental timeline. After 10-day cocaine self-administration and 7-day homecage withdrawal period, NAC (100 mg/kg) or VEH were administered intraperitoneally 30 min prior to extinction day 1. After 10-day extinction training and a subsequent 28-day homecage withdrawal period, rats underwent a spontaneous recovery test on day 55. **B** The mean number of cocaine infusions during self-administration acquisition (left) and the mean number of active and inactive nosepoke during extinction training (right). **C** Active and inactive nosepoke responses recorded during the last extinction training and the spontaneous recovery test. ***P < 0.001, ****P < 0.0001, comparing VEH to NAC. ○ and □ represent the performance of rats administered VEH and 100 mg/kg NAC, respectively. Active VEH = Active nosepoke in the VEH group. Inactive VEH = Inactive nosepoke in the VEH group. Last Ext Day = Last Extinction Day. SR test Day = Spontaneous recovery test. Data are expressed as the mean ± SEM. Details of the statistical analyses are shown in Supplemental Table 1.

Operant responding extinguished in the absence of reinforcement spontaneously recovers with the passage of time [40]. In Experiment 2, we further investigated whether NAC in conjunction with extinction training could suppress spontaneous recovery, thereby demonstrating its long-term therapeutic potential in preventing cocaine relapse. All experimental procedures were the same as those in Experiment 1, except that a spontaneous-recovery test was conducted on day 55 after the last extinction training (Fig. 2A). In this experiment, rats were randomly allocated to two groups and given a single dose of either NAC (100 mg/kg, i.p.) or VEH 30 min before the first extinction session. No significant differences were found in the total cocaine infusions during acquisition (Fig. 2B, left column). A two-way repeated-measures ANOVA revealed a significant interaction between treatment condition × extinction day on active nosepoke responses (*F*_*treatment* *×* *time*_(9,135) = 7.005*, P* < 0.0001). Post-hoc comparisons demonstrated that cocaine-seeking behavior was significantly lower in the NAC group compared to the VEH group on the first extinction day (Fig. 2B right column, *P* < 0.0001). Furthermore, a significant decrease in active nosepoke responses was observed during spontaneous cocaine relapse recovery in the NAC group compared to the VEH group (Fig. 2C left column, *F*_*treatment*_(1,15) = 7.849*, P* = 0.0134), while no differences were found in the inactive nosepoke (Fig. 2C right column). These results indicate that a single NAC injection paired with extinction training exerts long-term effects of anti-relapse, at least lasting 28 days.

The acute enhancement of extinction training observed on day 1 may result from multiple contributing factors, including locomotor impairments [41], potential analgesic effects [42, 43], and modulation of the glutamatergic system [44, 45]. Importantly, no differences were observed between groups during the subsequent extinction sessions; however, rats treated with NAC showed a marked reduction in cue-induced reinstatement. We further confirmed that the reduction in cocaine relapse induced by NAC was not due to nonspecific effects on locomotion, as no alterations in behavior were detected in the open field test (Supplemental Fig. 2). Hence, we suggest that NAC’s anti-relapse effect is mediated through modulation of the glutamatergic system, enhancing extinction learning specifically via activation of mGluR 2/3 receptors. To investigate this, a follow-up experiment was performed.

### Experiment 3: The long-term inhibitory effect of NAC on cocaine-seeking behavior depends on the extinction day 1 session and mGluR2/3-mediated glutamatergic signaling

**Fig. 3:**
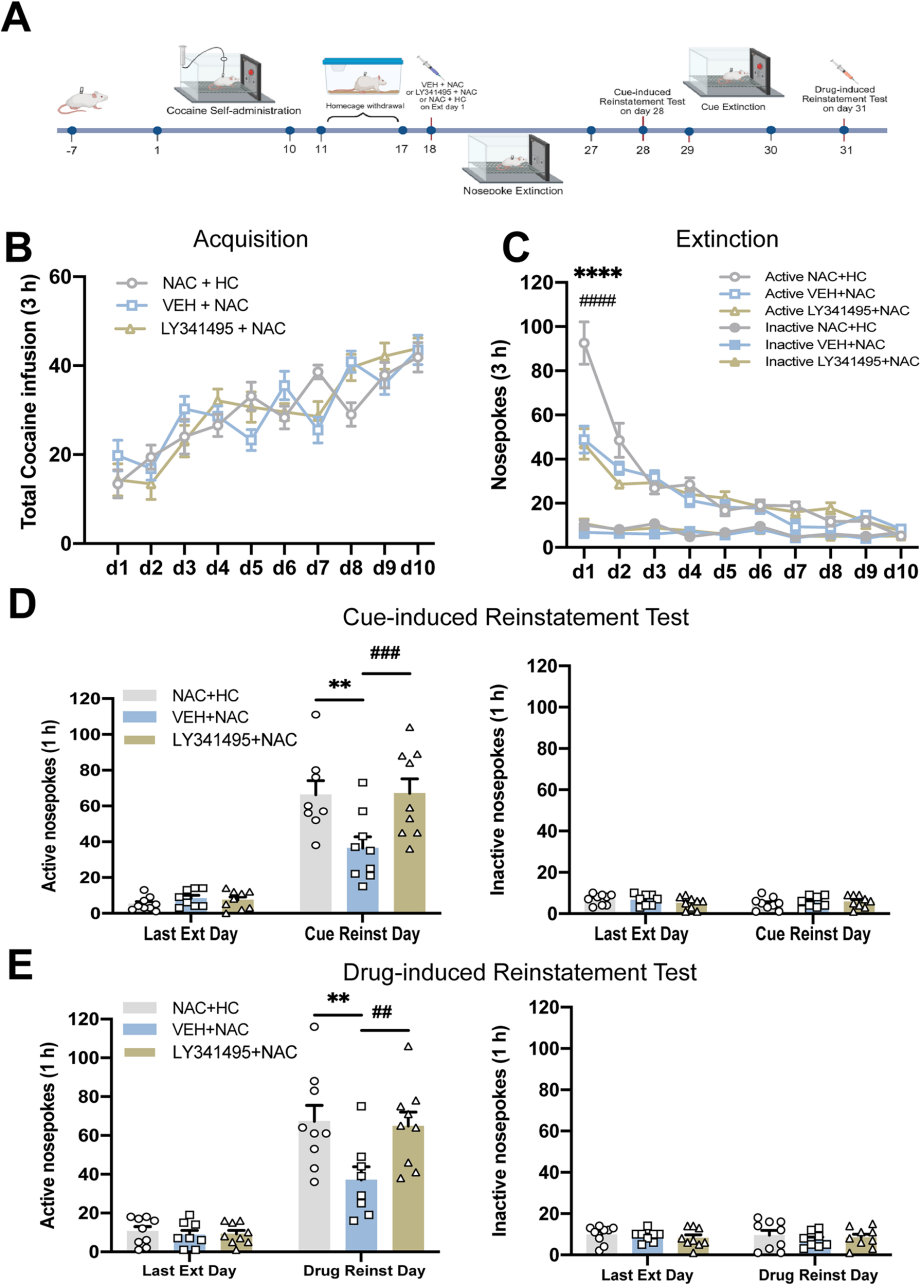
The long-term inhibitory effect of NAC on cocaine-seeking behavior depends on the extinction day 1 session and mGluR2/3-mediated glutamatergic signaling. **A** Experimental timeline. After 10-day cocaine self-administration and a 7-day homecage withdrawal period, rats received a single injection of NAC (100 mg/kg) 30 min prior to extinction day 1 or while in the homecage (NAC + HC). Extinction groups were additionally administered either LY341495 (1 mg/kg, i.p.) or VEH immediately before NAC treatment. **B** The mean number of cocaine infusions obtained during the acquisition of cocaine self-administration training. **C** The mean number of active and inactive nosepoke during the extinction training. **D** Active and inactive nosepoke responses recorded during the last extinction training and the cue-induced reinstatement test. ○, △, and □ represent the performance of rats in the NAC + HC, VEH + NAC, and LY341495 + NAC groups, respectively. **E** Active and inactive nosepoke responses recorded during the last extinction training and the drug-induced reinstatement test. **P < 0.01, ****P < 0.0001, comparing NAC + HC to VEH + NAC; ^##^*P* < 0.01, ^###^*P* < 0.001 ^####^*P* < 0.0001, comparing VEH + NAC to LY341495 + NAC. Active NAC + HC = Active nosepoke in the NAC + HC group. Inactive VEH + HC = Inactive nosepoke in the VEH + HC group. Last Ext Day = Last Extinction Day. Cue Reinst Day = Cue-induced Reinstatement Test Day. Drug Reinst Day = Drug-induced Reinstatement Test Day. Data are expressed as the mean ± SEM. Details of the statistical analyses are shown in Supplemental Table 1.

If the prolonged anti-relapse effect of NAC treatment observed in Experiment 2 results from enhanced extinction learning, it should rely on extinction training concurrent with NAC administration. In Experiment 3, rats were randomly allocated to three groups: (i) NAC + HC, which receiving NAC (100 mg/kg, i.p.) in the home cage rather than during extinction day 1 training, (ii) VEH + NAC, receiving a vehicle injection prior to NAC administration and underwent extinction day 1 training, and ((iii) LY341495 + NAC, receiving the mGluR 2/3 inhibitor LY341495 prior to NAC administration and extinction day 1 training (Fig. 3A). Similarly, total cocaine infusions during the acquisition phase did not differ significantly between groups (Fig. 3B). A two-way repeated-measures ANOVA indicated a significant interaction between treatment condition × extinction day for active nosepoke responses (*F*_*treatment* *×* *time*_(18,207) = 6.145*, P* < 0.0001). Post-hoc comparisons showed a marked reduction in cocaine-seeking behavior on the initial extinction day in the VEH + NAC group compared to the NAC + HC group (*P* < 0.0001), irrespective of mGluR2/3 receptor inhibition (Fig. 3C). Consistent with earlier findings, the groups showed no significant differences during the following extinction sessions. Nevertheless, a significant attenuation of cue- and drug-induced reinstatement was observed in the VEH + NAC group relative to NAC + HC or LY341495 + NAC groups (Fig. 3D, E, Cue-induced reinstatement, *F*_*treatment*_(2,46) = 4.525, *P* = 0.0161*;* Drug-induced reinstatement, *F*_*treatment*_(2,23) = 6.339*, P* = 0.0064). These findings indicate that the sustained suppressive effect of NAC on reinstatement requires both extinction learning and mGluR2/3-mediated glutamatergic signaling during NAC administration.

### Experiment 4: Pre-treatment with NAC prior to extinction day 1 also attenuates context-induced reinstatement of cocaine-seeking behavior

**Fig. 4:**
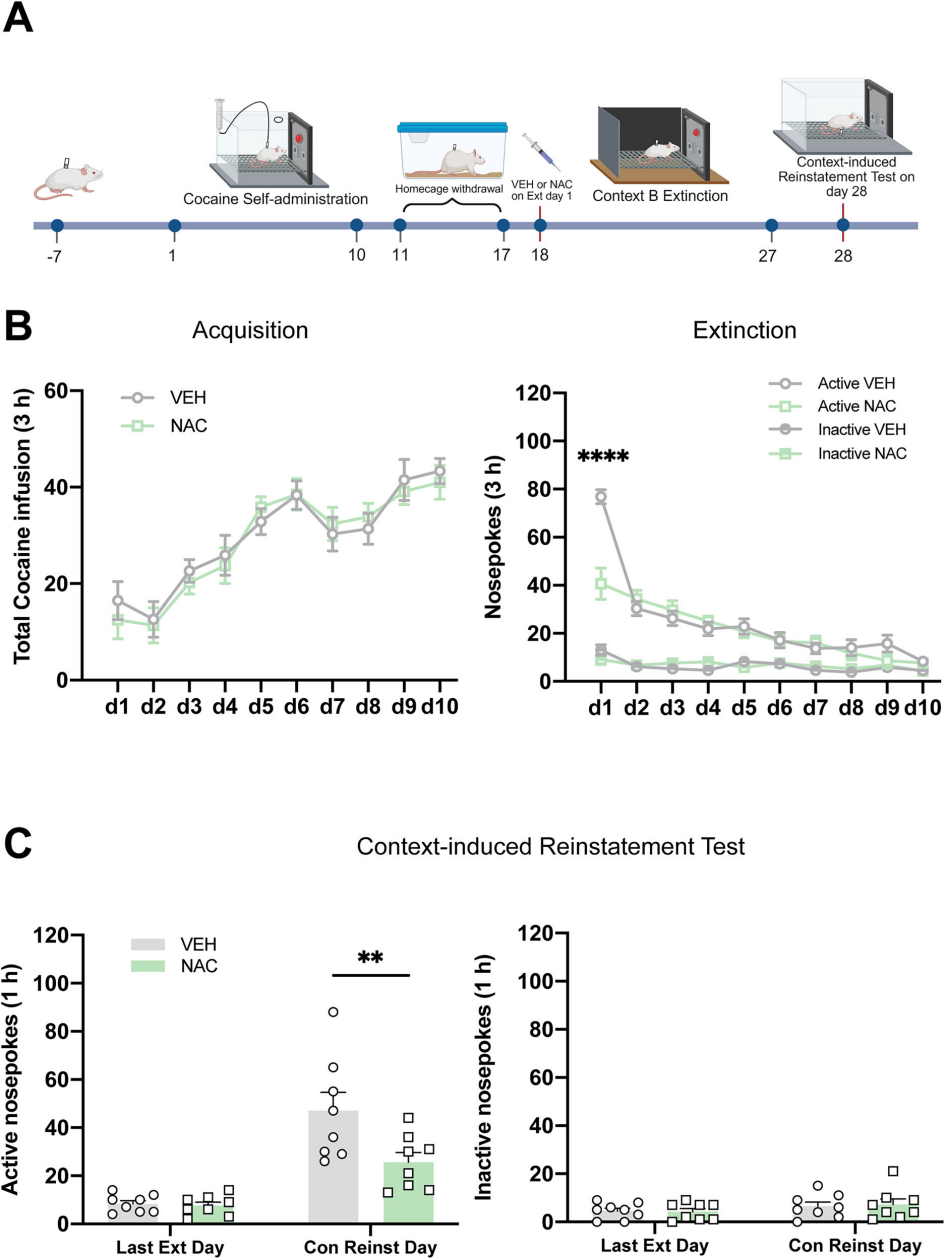
Pre-treatment with NAC prior to extinction day 1 also attenuates context-induced reinstatement of cocaine-seeking behavior. **A** Experimental timeline. The experimental design was identical to that of Experiment 1, except that extinction training was conducted in Context B, and rats underwent a context-induced reinstatement test on day 28. **B** The mean number of cocaine infusions during self-administration acquisition (left) and the mean number of active and inactive nosepoke during extinction training (right). **C** Active and inactive nosepoke responses recorded during the last extinction training and the context-induced reinstatement test. ○ and □ represent the performance of rats in the VEH and NAC groups, respectively. Active VEH = Active nosepoke in the VEH group. Inactive VEH = Inactive nosepoke in the VEH group. Last Ext Day = Last Extinction Day. Con Reinst Day = Context-induced Reinstatement Test Day. **P < 0.01, ****P < 0.0001, comparing VEH to NAC. Data are expressed as the mean ± SEM. Details of the statistical analyses are shown in Supplemental Table 1.

Considering that various factors contribute to drug-seeking behavior—including operant responses, discrete cues, and context [46], we next sought to determine whether the therapeutic effects of NAC extend to context-induced relapse. To test this, rats were randomly divided into two experimental groups: rats received a single injection of NAC (100 mg/kg, i.p.) or VEH 30 min before the first extinction. Rats underwent a 10-day extinction session in a distinct context B, followed by a context-induced reinstatement test conducted in the original training chamber (Fig. 4A). No differences were observed in the total cocaine infusions during acquisition (Fig. 4B left column). A two-way repeated-measures ANOVA showed a significant interaction between treatment condition × extinction day for active nosepoke counts (*F*_*treatment* *×* *time*_(9,126) = 7.033*, P* < 0.0001). Post-hoc analyses revealed that cocaine-seeking behavior on the first extinction day was significantly lower in the NAC group compared to the VEH group (Fig. 4B right column, *P* < 0.0001). During the context-induced reinstatement test, rats treated with NAC showed a marked decrease in active nosepoke responses relative to the VEH group (*P* = 0.0038), with no effect observed on inactive nosepoke responses (Fig. 4C). These results indicate that NAC’s therapeutic benefits generalize beyond discrete cues to include context, highlighting its potential to attenuate multiple forms of relapse-relevant behavior.

## Discussion

The present study demonstrates that a single systemic administration of NAC, when combined with extinction training, produces enduring suppression of cocaine-seeking behavior in rats. Specifically, NAC accelerated extinction learning on day 1 and subsequently reduced cue-induced reinstatement, drug-induced reinstatement, spontaneous recovery, and context-induced relapse of cocaine-seeking behavior. Importantly, the mGluR2/3 antagonist LY341495 reversed NAC’s effect on reinstated cocaine-seeking, demonstrating that activation of mGluR2/3 receptors is critical for the long-term therapeutic efficacy of NAC. These findings advance our understanding of NAC’s mechanism of action in addiction treatment and highlight its potential clinical utility as an adjunct to behavioral therapies.

### NAC timing and synergy with extinction: bridging acute and chronic effects

NAC, a clinically approved mucolytic agent with a well-established safety profile, has consistently shown therapeutic potential in substance use disorders through chronic regimens that sustained glutamate transporter modulation [47, 48]. Reichel et al. demonstrated that repeated administration of high-dose NAC (100 mg/kg) during extinction or abstinence produces enduring reductions in cocaine seeking [49]. In contrast, we demonstrate that a single NAC injection with the first extinction session also exerts long-lasting anti-relapse effects. This enhanced efficacy likely reflects two synergistic mechanisms. First, NAC facilitates the consolidation of extinction memory, enabling it to compete with original drug-related memories, thereby reducing subsequent relapse. Acute NAC administration has been shown to improve memory retention and cognitive performance across various tasks, including the Morris water maze, T-maze foot-shock avoidance, and memory consolidation in a conditioned place preference paradigm [50–52]. Its mechanism may involve an increase in endogenous glutathione, attenuation of oxidative stress, and enhanced synaptic plasticity [35, 53, 54]. Moreover, the systemic half-life of NAC is approximately 6.25 h [55], which aligns with the critical window for synaptic consolidation of around 6 h [56]. Second, NAC may exert its long-term behavioral effects by acutely restoring glutamate homeostasis and reversing cocaine-induced neuroplasticity that is required for reinstatement. In heroin models, it enhances GluA1 accumulation in dendritic spines in the nucleus accumbens [57]; in nicotine models, it reduces postsynaptic excitability and normalizes GLT-1 expression [58]. Cocaine exposure disrupts glutamate transmission by downregulating system xc^-^ and GLT-1 in the nucleus accumbens, leading to pathological synaptic potentiation and heightened relapse vulnerability [10, 31]. NAC rapidly increases extracellular glutamate via activation of system xc^-^, which helps normalize these neurochemical disruptions [44]. Critically, NAC suppresses relapse only when administered concurrently with extinction training, not during home-cage abstinence, highlighting that its therapeutic action requires re-exposure to drug-related cues. The result reinforces the idea that, rather than merely altering the baseline intensity or accessibility of previously established drug memories, NAC promotes the consolidation of new extinction memories. In summary, a single extinction-based treatment with NAC emphasizes a paradigm-shifting advantage for clinical translation—reducing treatment burden while enhancing compliance.

### mGluR2/3 dependence: a converging mechanism

NAC modulates glutamatergic homeostasis—via indirect mGluR5 agonism, mGluR2/3 presynaptic inhibition, and GLT-1 transporter upregulation—has prompted translational investigation into neuropsychiatric disorders with glutamatergic dysregulation [59, 60]. Experiment 3 identified mGluR2/3 activation as essential for NAC’s enduring effects, consistent with cocaine relapse studies [16, 61]. Moro et al. showed that acute NAC reduced cue-induced nicotine-seeking via mGluR2/3, an effect abolished by LY341495 pretreatment. Similarly, our data reveal that mGluR2/3 antagonism abolished NAC’s long-term—but not acute—suppression of cocaine-seeking (extinction remained accelerated, Fig. 3C). This dissociation suggests that while NAC’s immediate inhibition of drug-seeking may involve transient glutamate release (e.g., presynaptic mGluR2/3 activation to reduce synaptic glutamate), its enduring protection requires mGluR2/3-dependent consolidation of extinction memory. Notably, Reissner et al. found that NAC’s inhibition of cocaine reinstatement depended on restoring GLT-1 rather than via cystine-glutamate exchange [62]. While seemingly contradictory, these mechanisms may coexist: GLT-1 up-regulation could sustain extracellular glutamate homeostasis between extinction sessions, whereas mGluR2/3 activation during extinction trials directly suppresses cue-evoked synaptic glutamate release. Specifically, in the acute phase, NAC rapidly elevates extrasynaptic glutamate via cystine-glutamate exchange, which raises extra-synaptic glutamate levels to activate presynaptic mGluR2/3 autoreceptors, consequently decreasing synaptic glutamate release [45]. mGluR2/3 receptors have been implicated in reducing extracellular dopamine [63], which may partly underlie the inability of their antagonists to block NAC’s acute inhibitory effects. While in chronic/enduring phase: Sustained GLT-1 restoration maintains extracellular glutamate homeostasis, preventing spill-over onto extrasynaptic receptors (e.g., mGluR5) that potentiate relapse [64]. Our data highlight mGluR2/3 as the pivotal mediator of lasting effects, whereas chronic NAC regimens engage broader adaptations, including GLT-1 restoration.

### Clinical implications: toward a behaviorally-timed pharmacological strategy

The current findings offer important insights for clinical translation. While most previous trials have predominantly employed chronic, multi-dose regimens, yielding mixed results [64, 65]. In a clinical trial of adolescents with CUD, NAC doubled the rate of cannabis-negative urine tests relative to placebo after 8 weeks [66]. Although NAC has been associated with reductions in alcohol and nicotine use and modulation of inflammatory responses [67, 68], these effects have not been consistently replicated [69, 70].

Our results highlight a fundamentally different strategy: a single NAC dose timed to the commencement of exposure-based therapy. By aligning NAC delivery with the initiation of extinction learning, our approach capitalizes on a critical neuroplasticity window to enhance therapeutic outcomes. This markedly reduces the need for extended medication adherence—a significant barrier in addiction treatment—and minimizes potential side effects associated with long-term use.

Such a strategy aligns well with exposure-based interventions (e.g., cue-exposure therapy), which are widely used in the clinical setting but often limited by relapse due to inadequate consolidation of extinction memory. Furthermore, individuals with cocaine use disorder are unlikely to encounter environments comparable to laboratory extinction settings in clinical practice; augmenting extinction learning with a single adjunctive pharmacological intervention may help enhance the long-term efficacy of such behavioral therapies. As NAC is already FDA-approved for other indications and available over the counter, this approach offers exceptionally high translational potential compared to most experimental pharmacotherapies for CUD.

Our findings extend beyond traditional cue-induced relapse models. The observation that NAC reduced context-induced reinstatement—a potent trigger for human relapse—underscores its clinical utility in real-world settings, where environmental contexts are a primary precipitant of drug use. Therefore, this targeted, minimal-intervention strategy could offer a cost-effective, low-risk, and scalable adjunct to current treatments for cocaine addiction and potentially other substance use disorders characterized by cue- and context-dependent relapse vulnerability.

## Limitation

The present study has several limitations. First, only male rats were used, limiting the generalizability of the findings to females. Notably, NAC efficacy may differ by sex; for example, it failed to reduce cue-induced cocaine-seeking behavior in female rats [71]. Second, the paradigm was restricted to cocaine self-administration; thus, it is unclear whether similar outcomes would occur with other drugs of abuse [26]. Indeed, glutamatergic dysregulation is a shared hallmark of nicotine, alcohol, and methamphetamine use disorders. Methamphetamine exposure bidirectionally regulates glutamate receptor expression depending on exposure duration [72, 73], nicotine enhances AMPA and NMDA receptor expression on VTA dopamine neurons [19, 74], and alcohol disrupts glutamate clearance by downregulating GLT-1/xCT and glutamine synthetase [75, 76]. These convergent findings indicate common glutamatergic mechanisms across addictive substances, raising the possibility that the extinction training combined with the NAC approach described here could be extended to these drugs. Finally, mechanistic interpretations (e.g., presynaptic mGluR2/3 involvement and restoration of glutamatergic tone) were inferred from pharmacological manipulation rather than direct assay. Future studies should employ neurochemical or molecular techniques (e.g., glutamate microdialysis/quantification of transporter/receptor expression) to validate these mechanisms.

## Conclusion

A single NAC injection timed to the initiation of extinction training may harness the glutamate homeostasis mechanism—via system xc^−^ restoration and mGluR2/3 activation—to produce enduring suppression of cue- and context-driven relapse as well as spontaneous recovery. This non-invasive, pharmacological-behavioral strategy highlights NAC’s promise as an adjunct to extinction-based therapies and warrants further translational investigation.

## Supplementary information

Supplemental Figure 1

Supplemental Figure 2

Supplemental table 1

Supplementary information

## Data Availability

All data needed to evaluate the conclusions of the present study are presented.
