# Supplementary figures and images for: A single extinction-based treatment with N-Acetylcysteine produces long-term reduction in cocaine relapse
Source: Transl Psychiatry. 2026 Mar 19;16:186. doi: 10.1038/s41398-026-03954-2 (PMC13039927; doi:10.1038/s41398-026-03954-2)

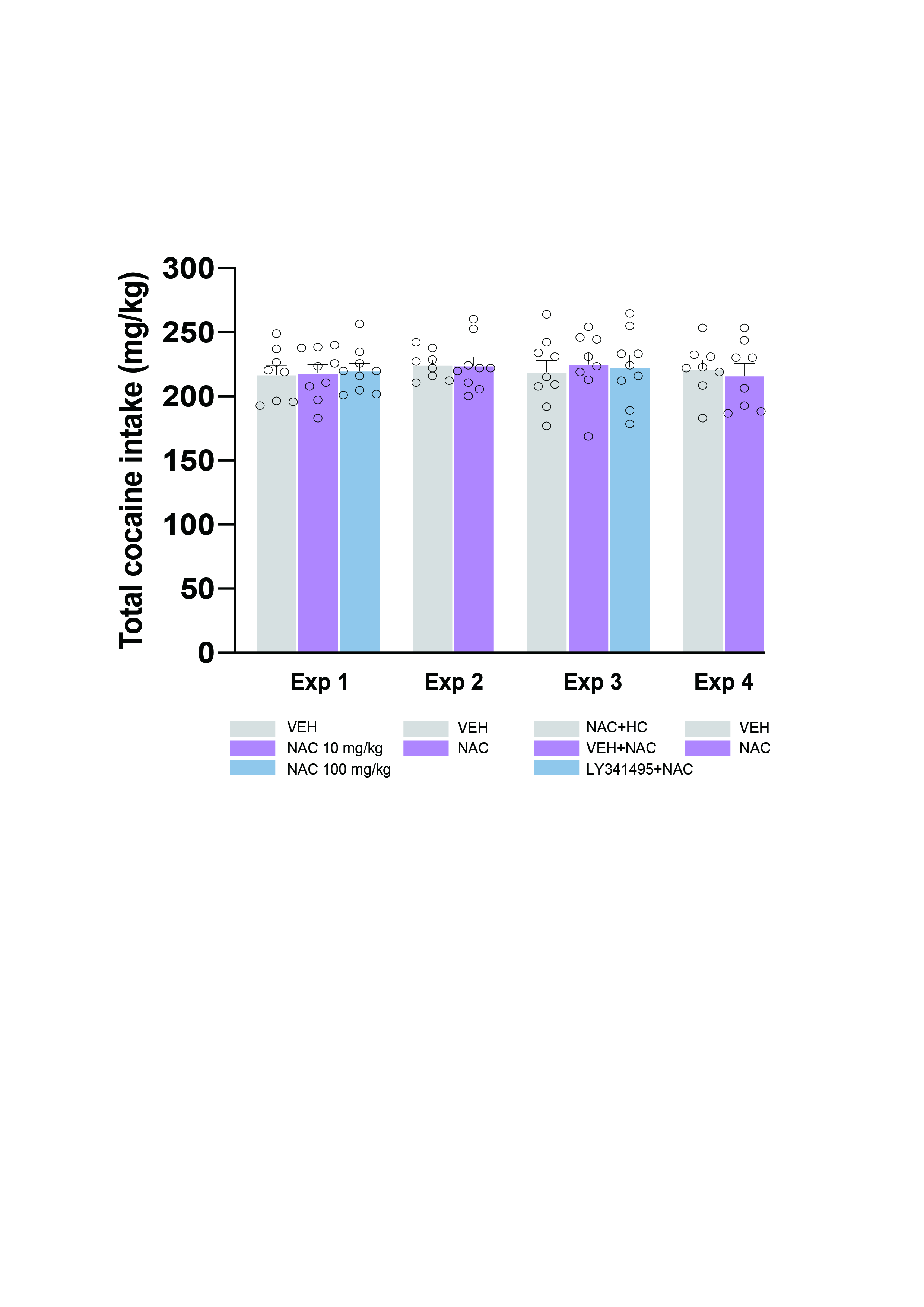

Supplement: Supplementary file 1 — Supplemental Figure 1 [file 41398_2026_3954_MOESM1_ESM.tif]

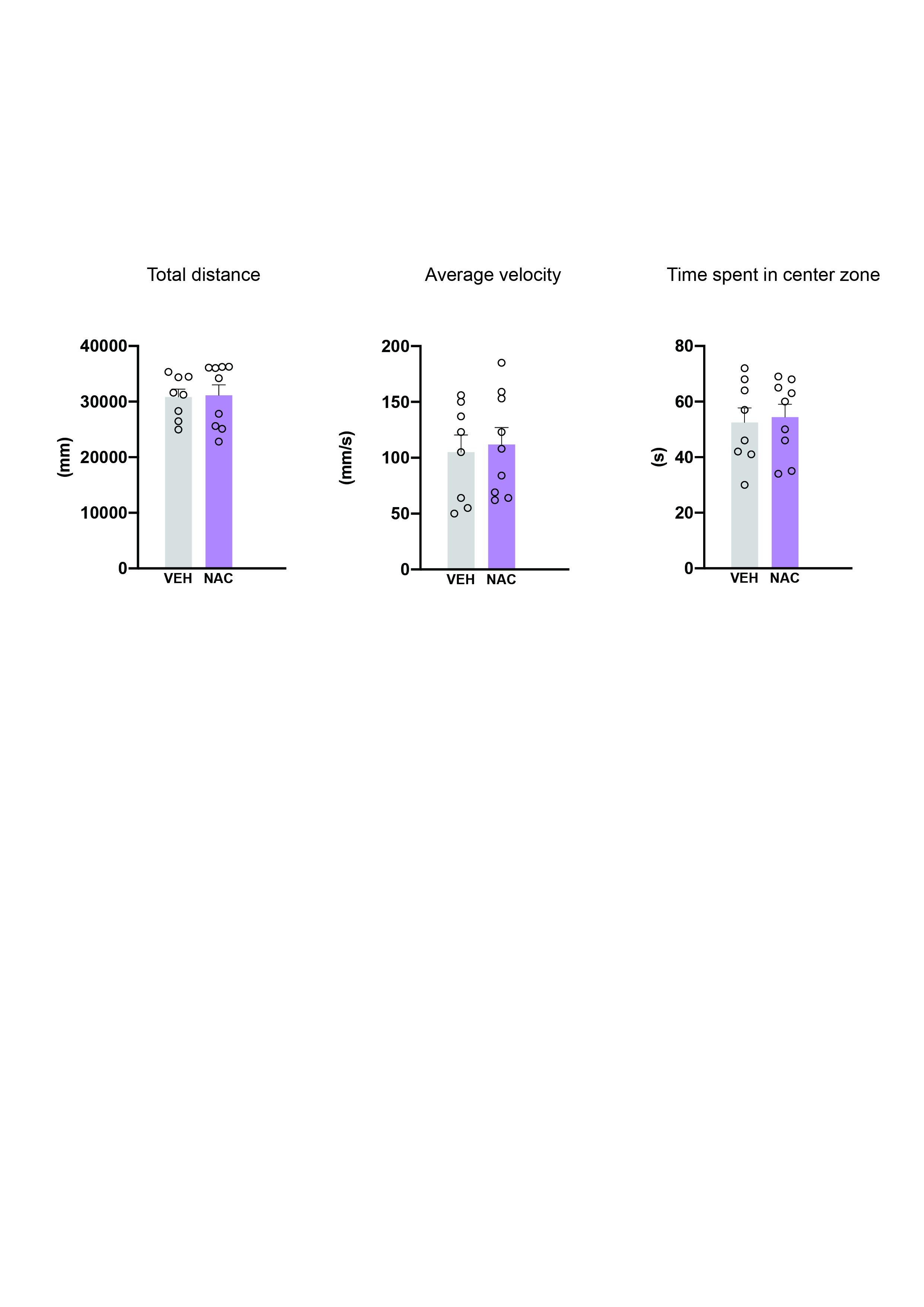

Supplement: Supplementary file 2 — Supplemental Figure 2 [file 41398_2026_3954_MOESM2_ESM.tif]
