# Supplementary material for: A single extinction-based treatment with N-Acetylcysteine produces long-term reduction in cocaine relapse
Source: Transl Psychiatry. 2026 Mar 19;16:186. doi: 10.1038/s41398-026-03954-2 (PMC13039927; doi:10.1038/s41398-026-03954-2)
Supplement: Supplementary file 4 — Supplementary information [file 41398_2026_3954_MOESM4_ESM.docx]

**Supplementary Figure 1. No significant differences in total cocaine intake across groups.**

Total cocaine intake during self-administration training in Experiments 1-4. Data are expressed as the mean ± SEM.

**Supplementary Figure 2.NAC had no effect on locomotor activity.**

Total distance, average velocity, and time spent in the central area during the open field test. Data are expressed as the mean ± SEM.
